# Accuracy and Sensitivity of Clinical Parameters in Predicting Successful Extubation in Patients with Acute Brain Injury

**DOI:** 10.3390/neurolint14030050

**Published:** 2022-07-25

**Authors:** Flávia Manhani Muzette, Rayssa Bruna Holanda Lima, Jennifer de Araújo Silva, Thamara Ferro Balsani Comin, Erlandson Ferreira Saraiva, Karla Luciana Magnani Seki, Gustavo Christofoletti

**Affiliations:** 1 Faculty of Medicine, Institute of Health, Federal University of Mato Grosso do Sul, UFMS, Campo Grande 79060-900, Brazil; flavia.muzette@gmail.com (F.M.M.); rayssa.lima_@hotmail.com (R.B.H.L.); erlandson.saraiva@ufms.br (E.F.S.); karla.magnani@ufms.br (K.L.M.S.); 2 Santa Casa Beneficent Hospital, Campo Grande 79002-251, Brazil; jenniaraujosilva@hotmail.com (J.d.A.S.); thamara.fbalsani@gmail.com (T.F.B.C.)

**Keywords:** brain injuries, neurological manifestations, critical care, intensive care units, mechanical ventilators

## Abstract

Background: Brain injuries are frequent causes of intubation and mechanical ventilation. The aim of this study was to investigate the accuracy and sensitivity of clinical parameters in predicting successful extubation in patients with acute brain injury. Methods: Six hundred and forty-four patients assisted at a high-complexity hospital were recruited. Patients were divided as for successful or failed extubation. The VISAGE score, maximum inspiratory and expiratory pressures, peak cough flow, and airway occlusion pressure at 0.1 s were used as predictors. Logistic regression analyses using ROC-curve identified values of accuracy and sensitivity. The Hosmer–Lemeshow test and the stepwise method calibrated the statistical model. Results: VISAGE score (odds ratio of 1.975), maximum inspiratory pressure (odds ratio of 1.024), and peak cough flow (odds ratio of 0.981) are factors consistent in distinguishing success from failure extubation. The ROC curve presented an accuracy of 79.7% and a sensitivity of 95.8%. Conclusions: VISAGE score, maximum inspiratory pressure and peak cough flow showed good accuracy and sensitivity in predicting successful extubation in patients with acute brain injury. The greater impact of VISAGE score indicates that patients’ neurological profile should be considered in association with ventilatory parameters in the decision of extubation.

## 1. Introduction

Acute brain injuries represent an important cause of hospitalization. These conditions affect the independence of patients and generate a prominent impact on public health [1,2].

Patients with acute brain injuries are frequently submitted to mechanical ventilation. Intubation usually occurs when airway protection is necessary and when patients’ intracranial pressure needs to be controlled [3,4]. The high prevalence of extubation failures in patients with acute brain injuries is an aggravating factor associated with tracheostomy, longer stay in the intensive care unit, greater hospital costs, and higher mortality rate [5,6].

Patient weaning usually passes through analyses of predictive factors associated with successful extubating. Up to now, there is no consensus guiding extubation of neurological patients [7,8,9]. Current parameters are not sufficiently clear to predict extubation success. Vital capacity, minute-ventilation, maximum inspiratory pressure, peak cough flow, spontaneous breathing trial, and the rapid and shallow breathing index are some of the factors used in choosing the right moment for extubation [9,10,11].

The use of ventilatory parameters without including neurological aspects might result in a patient’s reintubation [12]. While some studies recommend that extubation should be considered after reversal of the basic cause of the respiratory failure, others show that motor and cognitive sequelae can affect the airway protection capacity regardless of maintaining spontaneous ventilation [13,14,15].

Recent instruments, such as the VISAGE score, try to increase extubation success by including neurologic parameters in the decision to extubate [15]. In the present study, we investigated clinical parameters (ventilatory and neurologic) associated with successful extubation in patients with acute brain injuries.

We expect that this study may help health-care professionals in choosing the best moment for extubating patients with acute brain injuries, reducing the risks of reintubation, tracheostomy and death.

## 2. Materials and Methods

This is a descriptive and analytical study carried out with patients assisted in the intensive care unit of a high-complexity hospital. Ethical support was obtained with the Institution Review Board (protocol #3.723.230). Prior consent for participation in the research was collected from patients’ families before assessments.

The methodological procedures are described as recommended by the STROBE initiative [16]. Sample size was calculated considering the statistical design for logistic regressions, with type 1 error at 5%, power at 90%, and odds ratio of 3.6. The odds ratio was presented by a previous study about effective extubation in neurological patients [3]. The analysis indicated a minimum of 58 patients necessary to control type 1 (alpha) and 2 (beta) statistical biases.

**Figure 1:**
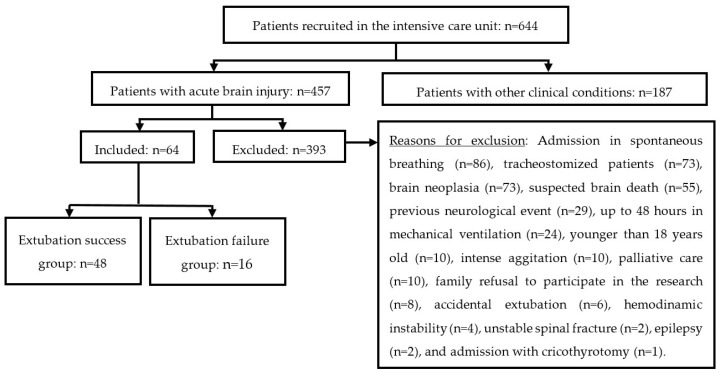
Flowchart of selection of participants.

Inclusion criteria involved patients diagnosed with acute brain injury, aged 18 years or more at entry, intubated, and under mechanical ventilation for more than 48 h in the intensive care unit. Brain injury was confirmed by clinical exam and head tomography. Exclusions involved pre-existing chronic brain injuries, patients with hemodynamic instability, intense agitation, presence of brain neoplasia, previous motor sequelae, individuals with suspected brain death, cases with spine fracture, epileptic state, tracheostomy, and those that have extubated themselves accidentally. Figure 1 details the flowchart of selection and follow-up of patients.

The recruitment of the subjects occurred in the first 24 h that the patient was referred to the intensive care unit. Researchers collected specific clinical information of each patient, such as level of awareness [17], severity index [18,19], presence of comorbidities, cause of the brain injury, cranial tomography, hospitalization time, sedation time, treatment and outcome.

After the patients’ awakening, ventilatory weaning was initiated. The extubation was based on the spontaneous breathing test [9,10]. Cases that required return to mechanical ventilation in less than 48 h were considered as extubation failure [19,20]. Before extubation, researchers assessed a series of predictive parameters aiming to perform accuracy and sensitivity analyses associated with successful extubation. Those variables are detailed as follow.

### 2.1. VISAGE Score

Developed by Asehnoune et al. [15], this score measures the extubation success of neurological patients based on age, presence of visual pursuit, swallowing attempt, and level of awareness. The summation of the factors in the VISAGE score predicts the extubation success rate, with higher values indicating better prognosis.

### 2.2. Respiratory-Muscle Strength

Respiratory-muscle strength was assessed by measurements of maximum inspiratory (MIP) and expiratory (MEP) pressures. A manometer (Indumed^®^, São Paulo, SP, Brazil) capable of measuring up to −120 cmH_2_O of MIP and +120 cmH_2_O of MEP was used. Inspiratory and expiratory muscle strength tests were performed according to recommendations suggested by Caruso et al. [21]. Previous studies confirm values higher than −20 cmH_2_O for MIP and +60 cmH_2_O for MEP to predict success in the ventilatory weaning of neurocritical patients [19,20,21,22].

### 2.3. Peak Cough Flow

This variable was included to analyze the patient’s cough effectiveness. Measurement was performed by the ASSESS Peak Flow Meter (Respironics^®^, Gray, ME, USA). The test was applied as recommended by Kutchak et al. [23]. In this assessment, values higher than 80 L/min predict extubation success.

### 2.4. Airway Occlusion Pressure at 0.1 s (P0.1)

This assessment was carried out through the Puritan Bennetttm 840 mechanical ventilator (Covidien Medtronic^®^, São Paulo, SP, Brazil). The analysis occurred as per the recommendation of Kera et al. [24], which predict values lower than 1.5 cmH_2_O indicating insufficient respiratory effort and values higher than 3.5 cmH_2_O suggesting exacerbated respiratory drive. The P0.1/MIP ratio was included in this study, as it is an integrative index for weaning success [25,26,27].

### 2.5. Statistical Analysis

The data analysis involved descriptive and inferential statistics. Regarding the descriptive statistic, continuous variables were expressed in mean and standard deviation (in case of parametric data) and median and interquartile range (in case of non-parametric data). Categorical variables were described in relative and absolute frequency.

The inferential analysis involved initially the independent Student *t*-test, Mann–Whitney U, and chi-square tests. Secondly, researchers performed logistic regression analysis to identify values of accuracy and sensitivity of successful extubation. The Hosmer–Lemeshow test was used to calibrate the statistical the model.

The VISAGE score, MIP, MEP, peak cough flow, and P0.1 were included in the regression models. The P0.1/MIP ratio was removed from the model to avoid multicollinearity bias. The stepwise method was used to identify significant variables in the statistical model.

Accuracy, sensitivity and the odds ratio were calculated to predict extubation success. The ability to discriminate successfully from unsuccessful extubations was quantified with the ROC curve. The statistical analyses were carried out in the programs Graphpad Software, Inc version 9 (Prism^®^, San Diego, CA, USA) and Software R version 4.0.2. (R Core Team^®^, Vienna, Austria). Significance was set at 5%.

## 3. Results

Six hundred and forty-four patients were recruited in this study. Five hundred and eighty patients did not meet the selection criteria (Figure 1). The number of patients enrolled was 10.3% higher than the minimal number of participants recommended in the sample size calculation. Participants’ anthropometric and clinical data are presented in Table 1.

Table 2 shows comparisons between groups in terms of VISAGE score, MIP and peak cough flow. Statistical differences between the success and failure extubation groups were seen for the VISAGE score (*p* = 0.031) but not for MIP (*p* = 0.085) and peak cough flow (*p* = 0.935).

Logistic regression analysis indicated significant interference of the VISAGE score, MIP, and peak cough flow on the extubation of patients with acute brain injuries. The VISAGE score and MIP showed positive coefficients in the logistic regression model. Peak cough flow, in contrast, had a negative coefficient. Table 3 details the coefficient of each variable in the logistic regression model.

Table 4 shows the odds ratio and the confidence interval for each of the explanatory variable. The results indicated that a one-unit increase in the VISAGE score increases the chance of extubation success by 75.42%. The increase by one unit of the MIP increases the chance of extubation success by 2.47%. The increase by one unit of the peak cough flow, in contrast, reduces the chance of success by 1.93%.

**Figure 2:**
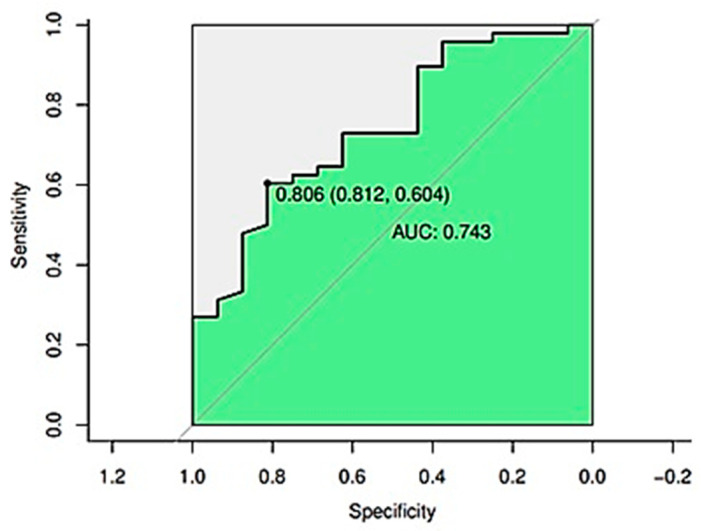
ROC curvature of the statistical model.

The adjusted model with the VISAGE score, MIP, and peak cough flow had an accuracy of 79.7%, a sensitivity of 95.8%, and an ability of predicting extubation success in 80.6% of the cases. Figure 2 shows the ROC curvature. Results confirm the capacity of the statistical model in controlling biases and identifying successful extubation.

In the failed extubation group, the main factors that culminated in the return of the patient to mechanical ventilation were loss of airway protection (56.2%), decreased level of consciousness (18.7%), laryngeal stridor (12.5%), acute respiratory insufficiency (6.3%) and hemodynamic decompensation (6.3%). Seventy-five percent of the patients that underwent failure in extubation were tracheostomized and twenty-five percent evolved into subsequent successful extubation.

## 4. Discussion

The aim of this study was to analyze predictive parameters associated with a successful extubation in patients with acute brain injury. Results were encouraging in demonstrating that VISAGE score, MIP and peak cough flow have great accuracy and sensitivity in predicting extubation success. The VISAGE score and MIP confirmed the authors’ hypothesis by demonstrating a direct relationship with a successful extubation. The peak cough flow, in contrast, diverged from the expected as they indicated an inverse relationship with extubation success. Understanding those parameters is essential to guide health-care professionals on the best moment to carry out extubation in patients with acute brain injuries.

This study selected patients with acute brain injuries assisted at an intensive care unit of a high-complexity hospital. Those patients are subject to large hospital costs and intensive treatments [28]. The procedure of ventilatory weaning and extubation occurred in all subjects, but with 25% requiring return to mechanical ventilation. The prevalence of failed extubation was similar as reported by Godet et al. [14], and Asehnoune et al. [15].

Considering that the extubation protocol was applied according to the spontaneous breathing test, which is commonly used in intensive care units and recommended in the literature [10,19,20], the authors recognized the need for including other clinical parameters capable of better predicting extubation success.

In this study, patients who had a successful or a failed extubation were similar to the anthropometric and clinical data (Table 1). This observation is important because if these variables interfered in patients’ outcome, they did it on a similar basis.

The only difference between groups occurred for days of hospitalization, where patients that had failed extubation stayed longer in the intensive care unit. This finding corroborates previous studies and can indicate that a longer hospitalization time can reduce success rates of extubation [29,30].

In the logistic regression analysis, the VISAGE score, MIP and peak cough flow were significant in predicting extubation success in 80.6% of the cases. This finding is important because neurological patients are subject to a series of complicating factors [5,6]. The results found in this study will enable a better conviction when opting for the extubation in neurological patients.

Among factors included in the statistical model, the VISAGE score showed the greatest potential for predicting success in extubation. This finding is encouraging as it confirms the need for including neurological state assessment and upper airway functionality, along with ventilatory parameters.

Table 2 demonstrates that patients with a successful extubation had a higher VISAGE score than patients with failed extubation. This result is close to that found by Asehnoune et al. [15], who identified a 90% success rate for extubation in patients with scores higher than 3.0 in that instrument.

In the present study, the logistic regression analysis confirmed that the increase in the VISAGE score is associated with a success rate of 75.42%. The authors attribute the success rate a little below the one found by Asehnoune et al. [15] to the VISAGE scores of patients in this study, slightly lower than the VISAGE scores of patients of that study.

Previous studies report a Glasgow Coma Scale score of less than 8 points is a prediction for extubation failure [6,31]. In this research, the success and failure groups showed a mean score of 11 points at the time of extubation (Table 1). This fact indicates that Glasgow Coma Scale may not be the most reliable predictor for performing weaning and extubation in patients with acute brain injury.

In fact, there is no consensus ensuring the use of Glasgow Coma Scale as a predictive factor to extubation in neurological patients [23,24,25,26,27,28,29,30,31,32]. Asehnoune et al. [33] attribute problems in the Glasgow Coma Scale to the fact that this instrument has not yet been validated, and, during intubation of neurological patients, the verbal command test cannot be performed by the patients.

As a way to analyze the level of consciousness of intubated patients, the authors proposed a series of simple motor tasks (such as opening and closing of eyes, carrying out of visual pursuit, and attempts to swallow) that enable the assessment of patient’s understanding and attention [33]. These tasks are present in the VISAGE score and the great potential that this instrument has as extubation success confirms that the analysis of patients’ consciousness must be incorporated in the clinical decision to extubate neurological patients.

The literature demonstrates divergences as to the use of MIP in predicting success or failure in extubation. While Freitas and David [34], and Kutchak et al. [23] observed differences in the MIP value of patients who had success relative to those who had failure in the extubation, Ko et al. [11] did not identify statistical significance of MIP to guide the extubation of neurocritical patients.

In the present study, both groups showed satisfactory MIP indexes, with a tendency for the successful extubation group to have a better score than the failed extubation group. The statistical model used in this research pointed out that a one-unit increase in MIP implied extubation success increase by 2.47%. This result may not be clinically relevant if we consider the impact of the VISAGE score, which is much more representative in the statistical model than the MIP.

The peak cough flow was included as a predictive factor considering prior studies that reported it as a factor of successful extubation [35,36]. Unlike the VISAGE score and MIP, peak cough flow showed different results as to extubation success. In our statistical model, a one-unit increase in peak cough flow implies decreased success in extubation by 1.93%.

This finding contradicts the initial hypothesis of the authors, who expected to find a direct relationship between peak cough flow and successful extubation. The authors justify this apparent contradiction by the satisfactory peak cough flow results seen in both success and failure extubation groups (Table 2). Because no significant difference was identified between groups for this variable (*p* = 0.935), the authors believe that the strength of this factor in the statistical model was reduced and thus brought results different than expected. New studies must be carried out to verify if the pattern found in this research is present in other situations.

Similarly, the authors expected that the P0.1 would be included in the final statistical model due to the precision of this variable as a marker of inspiratory effort [22,27]. Nevertheless, P0.1 values were similar between the success and failure groups and did not have significant impact to be incorporated into the logistic regression model by the stepwise model.

### Limitations

Although the current study provides important information about clinical parameters in predicting successful extubation in patients with acute brain injuries, it has some limitations that need to be considered. First, the results are restricted to intubated neurological patients under mechanical ventilation for more than 48 h in the intensive care unit. Second, the statistical model was not capable of predicting 19.4% of the successful extubations in neurocritical patients. While levels of accuracy and sensitivity were promising as to the use of the VISAGE score, MIP and peak cough flow, new studies must be carried out searching for other predictive factors capable of increasing extubation success.

## 5. Conclusions

The statistical model formed by the VISAGE score, MIP and peak cough flow showed a good accuracy and sensitivity in predicting successful extubation in patients with acute brain injuries. The greater impact of the VISAGE score indicates that a thorough neurological assessment must be incorporated into the analysis of ventilatory parameters before deciding to extubate the patient.

## Figures and Tables

**Table 1:** General characteristics of the participants.

Variables	Groups		*p*
	Success in Extubation	Failure in Extubation	
Sample size, n	75.0	25.0	0.001
Age, years	49.0 ± 19.0	58.0 ± 13.5	0.105
Sex, % (Male/Female)	70.9/29.1	81.2/18.8	0.424
Cause of injury, % (Clinical/Traumatic)	43.7/56.3	50.0/50.0	0.664
Level of awareness on patient’s admission, pts	8.5 ± 3.5	8.0 ± 3.5	0.737
Level of awareness at extubation, pts	11.0 (10.0–11.0)	11.0 (10.0–11.0)	0.912
Severity index, pts	21.5 ± 5.0	21.5 ± 6.0	0.893
Sedation time, days	3.0 ± 2.0	3.0 ± 1.5	0.713
Orotracheal tube time, days	7.0 ± 2.5	6.0 ± 2.5	0.283
Hospitalization time, days	9.0 ± 3.0	12.0 ± 4.5	0.002
Treatment, % (Conservative/Surgical)	41.6/58.4	43.7/56.3	0.884

Results are presented in mean ± standard deviation in parametric continuous variables, median (interquartile range) in non-parametric continuous variables, and percentage in categorical variables. *p* value of the Student *t*-test in parametric continuous variables. *p* value of Mann–Whitney U test in non-parametric continuous variables. *p* value of chi-square test in categorical variables.

**Table 2:** Performance of the VISAGE score, MIP, and peak cough flow in each group.

Variables	Groups		*p*
	Success in Extubation	Failure in Extubation	
VISAGE score, pts	2.5 (2.0–3.0)	2.0 (1.0–3.0)	0.031
Maximum Inspiratory Pressure, cmH_2_O	−89.0 ± 25.0	−77.0 ± 18.5	0.085
Peak cough flow, L/min	100.0 (76.0–160.0)	95.0 (70.0–120.0)	0.935

Results are presented in mean ± standard deviation in parametric variables and median (interquartile range) in non-parametric variables. *p* value of Student *t*-test in parametric variables. *p* value of Mann-Whitney U test in non-parametric variables.

**Table 3:** Estimates of parameters evaluated in the linear regression model.

Parameters	Estimate	Standard Error	*Z_value_*	*p*	95% Confidence Interval
VISAGE score	0.562	0.280	2.001	0.045	0.042 to 1.166
Maximum Inspiratory Pressure	0.024	0.011	2.154	0.031	0.003 to 0.048
Peak cough flow	−0.019	0.008	−2.196	0.028	−0.038 to −0.003

**Table 4:** Estimates of the evaluated parameters by odds ratio.

Parameter	Odds Ratio	95% Confidence Interval
VISAGE score	1.754	1.043 to 3.210
Maximum Inspiratory Pressure	1.024	1.003 to 1.049
Peak cough flow	0.981	0.962 to 0.997

## Data Availability

The data are available on request from the corresponding author.
